# Feasibility Study of Using Mobile Phone-Based Experience Sampling to Assess Drug Checking by Opioid Street Drug Users

**DOI:** 10.21203/rs.3.rs-2472117/v1

**Published:** 2023-01-13

**Authors:** James A. Swartz, Mary Ellen Mackesy-Amiti, A. David Jimenez, Lisa Robison-Taylor, Elizabeth Prete

**Affiliations:** 1 Jane Addams College of Social Work, University of Illinois Chicago; 2 Community Health Sciences, School of Public Health, University of Illinois Chicago; 3 Chestnut Health Systems, Chicago, Illinois

**Keywords:** drug checking, ecological momentary assessment, mobile ecological momentary assessment, opioid overdose prevention, experience sampling, fentanyl test strips, m-EMA

## Abstract

**Background:**

To date, evaluations of take-home fentanyl (and/or benzodiazepine) test strip use – the most common form of drug checking services – and potential effects on overdose risk have relied on retrospective accounts for some preceding time period, usually a week to several months. Such accounts, however, are subject to recall and memory biases. This pilot study assessed the feasibility of using experiential sampling to collect daily information *in situ* on drug checking and associated overdose risk reduction – the primary outcomes - among a sample of street opioid users and compared the results to retrospective reports.

**Methods:**

We recruited 12 participants from a Chicago-based syringe services program. Participants were 18 years of age or older, reported using opioids purchased on the street 3+ times per week in the past month, and had an available Android mobile phone. A phone-based app was programmed to collect daily drug checking information and provided to each participant along with a supply of fentanyl and benzodiazepine test strips and instructions for use over 21 days. Comparable retrospective data were collected via follow-up in-person surveys at the conclusion of daily report collection.

**Results:**

We found a reasonably high rate of daily reporting (63.5%) with participants submitting reports on 160 “person-days” out of 252 possible days. Participants submitted daily reports an average of 13 of 21 days. Reports of test strip use frequency varied between the retrospective and daily reports with a relatively higher percentage of days/time using test strips obtained from the daily reports. We also found higher proportions reporting overdose risk reduction behaviors on the daily reports compared with the retrospective reviews.

**Conclusions:**

We believe the results support using daily experience sampling to collect information on drug checking behaviors among street drug users. Although resource intensive in comparison to retrospective reports, daily reporting potentially provides more detailed information on test strip use and its association with overdose risk reduction and, ultimately, fewer overdoses. Needed are larger trials and validation studies of daily experience sampling to identify the optimum protocol for collecting accurate information on drug checking and overdose risk reduction behavior.

## Background

Drug checking services (DCS) consist of various methods by which street drug samples of unknown composition are tested to ascertain constituent psychoactive drugs as well as cutting agents. The information obtained through DCS can then be used to provide feedback to drug users on the composition of their illegally purchased/manufactured street drugs allowing the user to take precautions before using (e.g., taking a smaller dose than usual) to avoid or reduce the chances of an adverse health outcome such as an overdose. Depending on the checking method, the information provided can be limited to detecting the presence or absence of a single drug or as expansive as informing not only on the presence or absence of constituent drugs in a mixture as well as the amount/concentration of each drug detected. (1)

DCS as a harm reduction strategy was first developed in Europe in the 1990s where it was (and is still) used in nightclubs and social events to test for harmful adulterants in “party drugs” such as MDMA (i.e., “ecstasy”). (2) Owing largely to the promiscuous use of the highly potent synthetic opioid fentanyl and related analogs to produce illegally manufactured opioids as well as in admixture with other street drugs such as cocaine and MDMA (i.e., “Ecstasy”), and the consequent increase in drug-related overdoses and associated fatalities, there has been a renewed and growing interest in DCS as a prospective intervention for reducing both overdoses and fatalities. (3–7) Over the past 5 years, DCS methods have been adapted in Vancouver and elsewhere (e.g., Australia and Europe) as well as in Baltimore, Providence, San Francisco and other larger US cities to test street-purchased opioids and other drugs for the presence of fentanyl and analog compounds as well as common adulterants such as quinine, mannitol, and starches. (8–12)

Although sophisticated and accurate testing methods such as mass spectrometry and ion mobility spectrometry are available, they are expensive and require a high level of technical expertise beyond what is typically available in low and under-resourced syringe service programs (SSP) operating in community settings. (3, 12, 13) The use of these devices has therefore been restricted to testing locations where trained lab technicians are present such as at supervised injection sites or where it is feasible to transport samples for off-site testing. Consequently, simple, cheap, and less informative but still accurate means of drug checking such as fentanyl or benzodiazepine test strips have been more widely deployed in community settings and emergency departments as part of implementing DCS for street drug users. (14–17)

Fentanyl test strips were not initially designed to test drug samples directly but instead were intended for forensic purposes to test urine for the presence of fentanyl and analogues. However, because they are inexpensive (~$1 per strip), simple to use and read, and provide rapid results (~ 3 minutes), they have been adapted as a DCS for illicit opioids. Instead of dipping the test strip into urine, it is dipped into a solution of water mixed with a small amount of the street drug being tested. Recent research has demonstrated these test strips are accurate for detecting fentanyl and common analogues and have high levels of acceptability among opioid users as a form of drug checking. (10) Owing to the frequency with which benzodiazepines such as valium and alprazolam (e.g., Xanax) are being mixed into illegally manufactured opioids (17, 18), benzodiazepine test strips, which work similarly to fentanyl test strips, are now also being distributed to street drug users as a DCS component. (19)

Although barriers such as stigma and inconvenience can limit use of test strips for drug checking, evaluations suggest most street drug users are willing to employ them to test their drugs and, for many users, drug checking with test strips can lead to behavioral changes that reduce the probability of an overdose (e.g., taking a test dose or using less of the drug than initially planned). (10, 15, 20–22) To date, however, and to the best of our knowledge, evaluations of take-home fentanyl (and/or benzodiazepine) test strip use and potential effects on overdose risk reduction have relied almost exclusively on soliciting retrospective accounts from study participants for some preceding time period, usually a week to one or several months. (23–25)

As with retrospectively measuring any self-reported behavior or affective state that can change on a daily or even momentary basis, (e.g., cigarettes smoked, drug craving, depression or anxiety, number of drinks containing alcohol consumed) using retrospective measures to assess off-site drug checking with test strips can be subject to cognitive distortion and recall bias. (26, 27) For instance, more recent events or more striking events tend to unduly influence recollection of the entire recall period. There are additional context-specific limitations to using retrospective self-report to assess test strip use and its effects on drug use and overdose risk reduction. For instance, because illegal drug use is highly stigmatized, there is a tendency towards under-reporting. (28, 29) In addition, because of the parallel set of behaviors that can occur independently, concurrently, and/or repeatedly over time – using drugs, checking drugs, reducing overdose risk behaviors – using retrospectively recalled data makes it difficult to identify and compare specific occasions when users were more or less likely to have checked their drugs and occasions when drug checking did or did not lead to behaviors to reduce overdose risk. (30)

One alternative to using retrospective accounts of drug checking among street drug users is ecological momentary assessment (EMA). EMA involves a set of methods (e.g., daily diaries, experience sampling) whereby participants self-report information one or more times per day over some number of days, weeks, or even months in response to time-based prompts or when a specific event of interest to the study occurs. (31, 32) As data are collected *in situ* and in near real-time from study participants, EMA methods have the potential to reduce recall bias by more proximally aligning measurement of the variable(s) of interest with the time and place of their occurrence. EMA also allows for greater discernment of the dynamic associations among study variables owing to the repeated measurements over time and occasions. (30) Although EMA methods have been successfully used to study a variety of health-related issues including substance use and misuse (33–38), to the best of our knowledge, there are presently no published peer-reviewed studies using EMA methodology to assess the effectiveness of drug checking using take-home test strips as a harm reduction strategy for reducing illegal drug-related overdose risk.

The present study sought to assess the feasibility of using experience sampling to collect information on drug checking and overdose risk reduction among a sample of street opioid users accessing services at a community-based SSP. In particular, we wanted to determine the extent to which participants would be willing and able to provide daily reports on the following patient reported outcome measures (PROMS): drug use, drug checking using test strips, and overdose risk-reduction efforts made based on the drug checking results received. We also wanted to assess how burdensome or easy they found using a mobile EMA (mEMA) system whereby prompts and reporting forms were delivered via a mobile phone-based application and how difficult or useful they found testing their drugs with the provided test strips. Last, we wanted to compare retrospective reports on frequencies of drug checking, overdose risk reduction behaviors, and overdoses experienced with estimates based on the experience sampling data.

## Methods

### Setting

Feasibility study data were collected during the third phase of a three-phase research project that also included phases to assess current attitudes towards drug checking and develop a novel take-home drug-checking method - the illegal drug Paper Analytic Device (idPAD). (39–41) Recruitment for each phase was conducted independently to obtain a unique sample. The study protocol was reviewed and approved by the UIC institutional review board.

For all study phases, we recruited participants from among clients of a Chicago-based SSP that provides harm reduction and health care services to street drug users. Based on a larger sample (N = 124) recruited during a prior phase, the client population is composed predominantly of ethnic minorities (59.5% African American/ Black, and 21.9% Latinx), males (78.5%), has a mean age of 48.3 years, lives in socio-economically disadvantaged communities on Chicago’s west and northwest sides, and has a past-year homeless rate of 31.5%. (42) SSP clients reported using heroin an average of 27.6 days and fentanyl 22.7 days in month prior to study enrollment. Just over 45% of SSP clients sampled in 2020-2021 indicated they experienced 1 or more past year opioid-related overdoses.

### Sample

We recruited a convenience sample of 12 participants from SSP clients between April and June 2022. Study inclusion criteria were: 1) 18 years of age or older; 2) current and frequent opioid user defined as using 3 or more times in a typical week; 3) ability to comprehend informed consent and understand conversational English; and 5) access to an Android-based mobile phone they were willing and able to use for the study.

### Measures

#### Demographics.

At baseline, we collected demographic information on gender identity, sexual identity, race/ethnicity, age, housing status/homelessness, and income level.

#### Serious Mental Illness (SMI).

Participants were also asked a series of six questions about past-month symptoms of psychological distress indicating SMI using the K6 screening scale. We used an established threshold of > 13 to indicate SMI. (43–45)

#### Retrospective Reports on Substance Use, Drug Overdoses, and Overdose Risk Reduction Precautions.

At baseline and again at follow-up, we asked a series of questions on past-month substance use. These included questions on which substances were used and number of days used in the past month for 14 different drugs that included: heroin, fentanyl, prescription opioids, methadone, buprenorphine, and cocaine. We also asked participants at baseline the number of lifetime (up to 100) and past-year drug overdoses experienced and any steps they might have taken in the past month to reduce overdose risk based on a list of options derived from the most common risk reduction precautions reported by SSP clients in the first study phase. The list included: decreasing the amount of drug taken; using with others present; administering the drug more slowly than usual or first administering a small test dose; and/or having naloxone available before using.

#### Daily Experience Sampling of Drug Use, Drug Checking, and Overdose Risk Reduction Precautions.

As we could not identify prior studies of using experience sampling to assess drug checking, we adapted questions from retrospective surveys we administered in the other phases of the study. The daily mobile phone app survey asked participants to report on whether they used any street drugs that day, if so whether they checked the drug for fentanyl or benzodiazepine using the provided test strips, and whether they took any overdose risk precautions as a result of the drug checking results. If they did not purchase and/or use any street drugs that day, no further questions were asked. If the participant reported purchasing and using street drugs that day but did not pre-test them, they were asked the main reason(s) they did not test their drugs after which the survey for that day ended.

If they did pre-test their street drugs using the test strips, they were asked what the drugs they purchased were sold as and if the testing yielded positive results (yes/no/uncertain). Given they indicated obtaining a positive fentanyl or benzodiazepine test result, they were next asked what precautions they took (if any) to reduce their overdose risk. Finally, using 10-point Likert scales, the daily survey asked those who used a test strip that day how they would rate the ease/difficulty of using the fentanyl test strip(s) and how clear and easy to understand the results were. Two additional questions using the same 10-point Likert scales assessed ease/difficulty and clarity of results for the benzodiazepine test strips.

In addition to the daily report data provided by the participants, the system automatically recorded the date and time of the survey; the times the survey was begun and completed; and the time the daily reminder was sent. We used these time/date stamps to eliminate responses that appeared to be duplicates owing to a short time between submissions (i.e., < 1 hour).

#### Event-driven Overdose Reporting.

Information on any drug-related overdoses experienced during data collection was also obtained via the mobile phone app but without prompting. The overdose survey form included questions asking for the date and time of the overdose; whether they were with someone else such as an injection partner/friend, stranger, acquaintance, or someone else when the overdose occurred; whether naloxone was administered; and if medical attention was received, including at an emergency department or hospitalization.

#### Retrospective and Daily Report Comparisons on Drug Checking Frequency, Frequency of Overdose Risk Reduction Precautions, and Test Strip and Experience Sampling App Use.

We used the information collected at a one-month follow-up to assess for differences between retrospective reports of drug checking frequency and overdose risk reduction methods undertaken and comparable results based on the daily experience sampling data. The follow-up survey asked participants to indicate what percent of the time they checked their drugs before use using a 4-category response option (0-24%, 25-49%, 50-99%, 100%). To obtain a comparable measure based on the experience sampling, we calculated the percentage of times a participant reported using a street drug and testing that drug before use and then created a 4-category outcome by dividing the number of fentanyl tests done by number of days used. We did not count days where a drug was not used or where the person reported they did not test because they had previously tested the drug used that day. We then collapsed the results into the same 4 categorical response options used in the follow-up survey.

To compare daily reported risk reduction methods with the retrospective accounts at follow-up, we tallied the number of daily reports that indicated use of each risk reduction method for each participant to obtain an estimate of frequency of that risk reduction method and then collapsed the total for each participant and method into a dichotomous (0/1) variable indicating whether a specific risk reduction method had been used or not used during the experience sampling data collection method.

The follow-up survey included questions about the perceived overall usefulness of the test strips for drug checking and the ease or difficulty completing the daily surveys and ease of using the mobile phone app using the same 10-point Likert scales as in the daily surveys. We compared these retrospective responses with the average response to the same questions asked at the conclusion of each daily survey where a participant reported using a test strip.

### Procedures

Prior to recruitment we conducted a pre-test of the app to assess the performance of the app and the clarity of the instructions provided. Three SSP clients participated in the pre-test, following which we made final minor adjustments to the app and instruction set.

Recruitment was done through posted fliers at both SSP locations and through program staff word-of-mouth. Program staff used a script to provide interested and eligible SSP clients with a description of the study and what involvement entailed. Those who remained interested were referred to an RA, who reviewed the study requirements in more detail, reassessed eligibility, obtained written consent and began the study by administering the baseline interview on a laptop computer using REDCap, a secure, web-based software platform designed to support data capture for clinical research studies. (46, 47) Following the baseline interview, the RA downloaded and installed a copy of the programmed movisensXS app onto the participant’s phone and went through a practice session as to how daily notifications would occur and how to fill out and submit the end-of-day reporting form as well as how to fill out and submit a form in case of an overdose. (48)

We used movisensXS v. 1.4.8, a mobile app that runs on Android devices, and which was designed to collect experience sampling data. (48) Prompts can be programmed in the app to elicit time or event driven responses via forms that are programmed into the app for a specific project. Data are collected with or without an Internet connection and uploaded to a secured server once a connection has been established. At the end of data collection, the information collected on the server can be downloaded as an Excel or ASCII file for further analysis.

We used both interval (daily surveys on test strip use) and event-driven (additional reports on any overdoses experienced during the collection period) sampling. Copies of both the daily and overdose surveys are included with the supplemental materials. We programmed the movisensXS app to send one daily reminder to fill out an end-of-day report at 6:00 PM each day to any participant who had not already submitted a report that day. End-of-day prompts were issued through a 10 second sound/vibration followed by a screen display for 50 seconds. Participants had an option to dismiss the alarm for up to 1 hour and complete the form later. We did not prompt for overdose reporting but instead instructed participants at the baseline interview on how to fill out this form if they experienced an overdose during the data collection period.

The RA provided each participant with an initial batch of 10 fentanyl and 10 benzodiazepine test strips with instructions to return to the SSP for additional test strips as needed. The RA then reviewed how to correctly use and interpret the test strip results and demonstrated their use during a practice session. Finally, each participant was provided with take-home informational forms (see materials included as supplemental) that reviewed how to use the app and provided contact information for reaching the RA if they encountered any difficulties or had questions during data collection.

Following the baseline interview and training session, the RA monitored participant responses over the 21-day data collection period by using the data stored on the moviesensXS server. Attempts were made to contact any participant who missed more than 3 days of reporting to determine if they were having difficulties that precluded them from participating and to offer corrective actions (e.g., reinstalling the app, reviewing use of the app, providing new test strips if the originals were lost) when possible. At the conclusion of the 21-days the RA scheduled the final follow-up interview to occur as soon as possible.

Participants received $30 for completing the baseline interview and $35 for completing the follow-up interview. They also received an additional $1/day for each of up to 21 daily survey summaries submitted with a $20 bonus for completing at least 18/21 daily surveys. The total possible compensation for completing all tasks was $106.00.

### Analyses

All analyses were conducted using Stata v.17.1 statistical software. (49) Graphics were created using the ‘ggplot2’ package running under the R v.4.2.2 programming language. (50, 51) We exported the experience sampling data from the movisensXS server and uploaded it into the REDCap project where we combined it with the baseline and follow-up survey data for the analyses. Because of the limited sample size and corresponding lack of statistical power, we did not estimate inferential statistics or comparisons but instead focused on descriptive analyses.

We calculated demographic information – frequencies, percentages, standard deviations, and means – using the baseline survey data. We then summarized the daily experience sampling data to show both the total number of daily reports submitted by participant and the total number of reports submitted on each day of the 21-day data collection period. We next calculated the conditional percentages of participants who met each step over a cascade of contingent steps that began with testing their street drugs prior to use each day, to obtaining a result positive for fentanyl given testing was done, to taking precautions to reduce overdose risk given a positive test result. We also summarized the explanatory information at each step explaining why the next step was not undertaken (e.g., why testing was not done on a given day despite drug use) to better understand the barriers to using test strips and, ultimately, taking overdose risk precautions.

Finally, we compared the data reported in the follow-up survey on overdose precautions taken over the past month, the ease and clarity of using the fentanyl test strips, and number of past-month overdoses with comparable metrics based on the summarized experience sampling data. As one participant did not provide follow-up survey data, we excluded that participant’s experience sampling data from these analyses. Last, we provide summary information on participant’s perceptions of the ease of use of the mobile phone app.

## Results

### Demographics and Baseline Drug Use

The feasibility study sample of twelve participants was composed of a smaller percentage of males and minorities, was somewhat younger, and less likely to be homeless than the SSP client population estimates obtained from our larger, prior study phase (see the Setting section above). Nevertheless, baseline demographic information indicated this sample also had a very high rate of homelessness, used heroin and/or fentanyl on a near daily basis, had incomes at or under the federal poverty level, and had a high rate of co-occurring serious mental illness. Just about sixty percent (58.3%) self-identified as male; 33% were from an ethnic minority group (16.7% African American/Black, and 16.7% Latinx); and had a mean age of 40.7 years. Fifty percent reported being homeless at any time in the past month with seventy five percent reporting an annual income of $19,999 or less. One-third of the sample reported symptoms consistent with an SMI in the past month per their K6 screening scale question responses.

Participants reported using heroin an average of 26.3 days in the past month, a prescription pain killer on 10.2 days, and *intentionally using* fentanyl or another synthetic opioid an average of 15.4 days. All twelve participants indicated they had injected drugs in their lifetime with eight (66.7%) reporting any past-month injection use. Ten of the twelve participants (82.3%) had experienced one or more overdoses in their lifetime (range = 1 – 19) with two participants reporting they had experienced an overdose in the past year.

### Daily Reports Submitted and Reporting Rates

**Figure 1:**
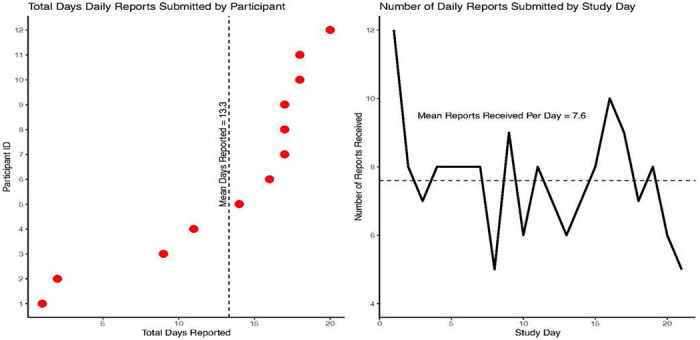
Total Number of Daily Drug Checking Reports Submitted by Participant and Study Day Note. All figures are based on the daily drug testing reports submitted over the 21-day study period by the 12 pilot test participants. The left panel shows the total number of unduplicated reports submitted by each participant ordered from the fewest to the most reports submitted. The right panel shows the number of unduplicated reports received each day during data collected across all participants. Multiple reports submitted on the same day were counted as one report for that day. The dashed line in the left panel shows the mean days reported and in the right panel shows the mean number of participants reporting at least once per day.

As recorded by the mobile app, the average completion time per report was about one minute (mean = 52.9 seconds, range = 2 – 313 seconds, sd = 59.6). Figure 1 shows the total number of days a daily report was submitted by participant (left pane) and the average number of participants submitting at least one report per day (right pane) over the 21-day data collection period. On average, participants submitted at least one report on 13.3 days (range = 1 – 20; 95% CI = 9.3 – 17.4) yielding an overall reporting rate of 63.3% (i.e., reports submitted on 13 (61.9%) of the 21 days possible). However, excluding two clear outliers — participants who submitted forms on only 1 or 2 days — the mean increases to 15.7 days per participant (range = 9 – 20; 95% CI = 13.3 – 18.1) and the reporting rate to 71.4%. Aside from an initial sharp drop in reports submitted over the first 2-3 days and another dop on the final reporting day, participants were fairly consistent in filing at least 1 report per day over the 21 days of data collection. We did not observe, for example, a steady decline in the number of reports received over time.

### Test Strip Use, Testing Results, and Effects on Overdose Risk Reduction Behaviors

**Figure 2:**
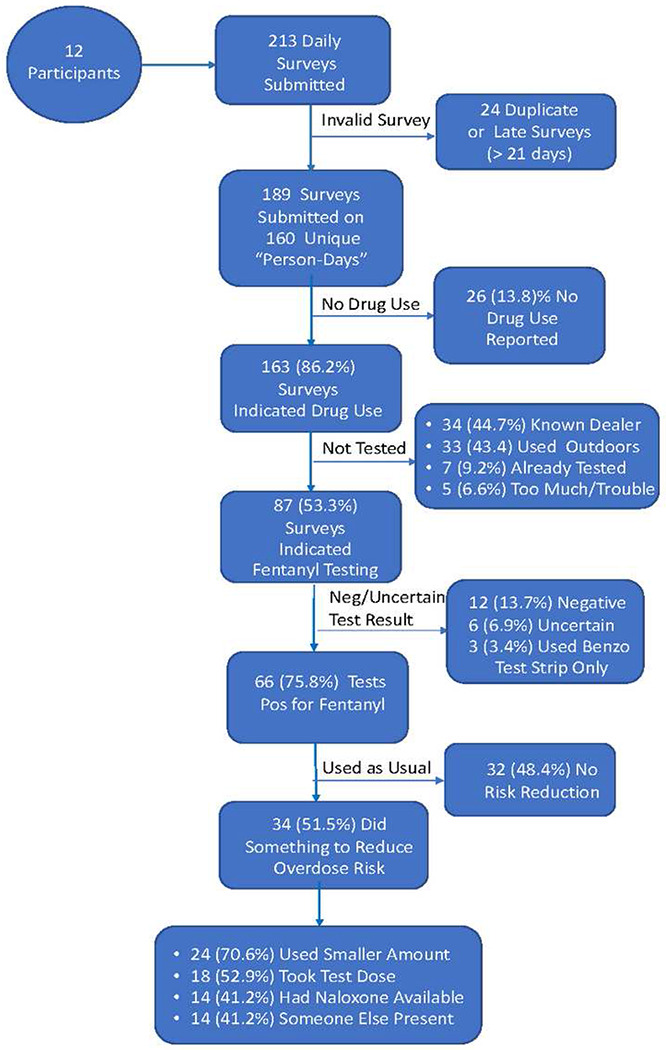
Flow Chart of Daily Test Strip Results from Submission to Risk Reduction Post-Testing Note. All figures are based on the number of valid daily reports submitted and/or the results obtained for drug checking at each reporting/testing step. All percentages shown are conditional, contingent on the number of reports submitted, the results of the submitted reports, or the behaviors taken given the obtained testing results. Because only a small number of tests (3) were conducted exclusively using the benzodiazepine test strips, the positive results and overdose risk behavior percentages are based on the reports of fentanyl test strip use.

Details on test strip use, reasons for non-use, and effects on overdose risk reduction behaviors are shown in the form of a flow chart (Figure 2). A total of 213 daily report forms were submitted of which 24 were deemed invalid as they were submitted within 1 to 60 minutes of the first form submitted that day or were submitted after the 21-day collection period ended for that participant.

The remaining 189 valid surveys were submitted on 160 unique “person-days” or 63.5% of the total possible 252 person-days (i.e., if all participants had submitted a daily report form every day). On 26 of the submitted daily surveys (13.8%), the participant indicated they did not use drugs on that day, concluding the report; 6 of the 12 respondents submitted at least one report of no drug use that day. Of the remaining 163 reports filed, 76 (46.7%) were marked as not using a test strip before using their drugs. Of the 76 reports indicating the drugs were not tested before using, 33 reports (43.4%) said the reason for not checking was that drugs were used outdoors in a public place; 34 (44.7%) reports said they had previously bought from the same dealer; 7 (9.2%) used from a previously tested supply; and 5 (6.6%) said testing was too much trouble and they did not want to wait. Only 3 (4.0%) reports said they did not have their testing supplies with them. No participants reported they did not test their drugs prior to use because they had run out of test strips.

Of the 87 daily reports indicating the drugs had been tested prior to use, 39 (44.8%) said the participant tested with a fentanyl test strip only; 45 (51.7%) reported testing with both a fentanyl and a benzodiazepine test strip; and on only 3 (3.5%) daily reports the participant said they tested solely with a benzodiazepine strip. On the 87 occasions drugs were tested prior to use, 66 (75.9%) were sold as heroin; 28 (32.2%) were sold as fentanyl; and 5 (5.7%) were sold as cocaine (more than one drug could be checked). Of the 84 occasions drugs were checked with a fentanyl test strip, 66 (78.6%) yielded a positive result, 12 (13.7%) were negative and 6 (3.4%) indeterminate. Interestingly, 2 of the 5 (40%) times drugs sold as cocaine were tested, the result was positive for fentanyl supporting reports that fentanyl is now being mixed with drugs other than opioids.

Participants were asked if they did anything to reduce their overdose risk following a positive result for fentanyl. About half of the reports (48.4%) indicated no change in drug use with the remainder of the reports (N = 34, 51.5%) indicating one or more steps was taken to reduce an overdose risk: 24 (70.6%) used a smaller amount than usual; 18 reports (52.9%) indicated a smaller test dose of the drug was taken or the drug was used more slowly; 14 (41.2%) said the drugs were used with someone else present; and 14 (41.2%) said they made sure naloxone was available.

#### Daily Reporting Mobile App Ease of Use.

Ten of the eleven participants who provided follow-up survey responses rated the mobile phone app as being very easy to use to report on their drug use and drug checking. Seven of the eleven gave the maximum score of 100 on the response scale and three other participants rated the phone app ease-of-use as 90 or 95.

#### Retrospective Report and Summarized Experience Sampling Data Comparisons Test Strip Ease of Use and Interpretation.

Responses collected and averaged from the daily surveys on test strip ease of use and interpretation were also very positive and consistent with the follow-up survey responses (Table 1). On the daily surveys, participant responses indicated both the fentanyl and benzodiazepine strips were easy to use and the results easy to interpret. For the fentanyl test strips, the mean ease of use score was 8.5 (range = 6 – 10; sd = 1.5) and the mean clarity of results score was 8.4 (range = 6 – 10; sd = 1.7) of 10 possible points. Scores for the benzodiazepine test strips were only slightly lower but still indicated the strips were easy to use and interpret; mean benzodiazepine ease of use score = 8.0 (range = 6 – 10; sd = 1.7), mean benzodiazepine ease of interpretation score = 8.0 (range = 6 – 10; sd = 1.5). The corresponding follow-up survey ratings, where only fentanyl strip use was asked about, while somewhat lower and more variable for fentanyl strip ease of use (mean = 7.2; range = 3 – 10; sd = 2.3) were still very positive as was ease-of-interpretability (mean = 8.9, range = 5 – 10; sd = 1.8).

#### Fentanyl Test Strip Use.

Per both measures, a high proportion of participants used fentanyl test strips in the past month; 90.9% on the follow-up survey and 100.0% submitted at least one daily report indicating test strip use. There was a greater discrepancy between the follow-up and daily reports in terms of the percent of time a fentanyl test strip was used with the daily reports yielding higher testing rates. The main difference was that 45.5% of the daily reports submitted when a street drug was reported used that had not been previously tested indicated a 100% testing rate whereas none of the follow-up surveys indicated the same testing rate. Instead, 30.0% of the follow-up surveys indicated greater than 50.0% of the time but less than 100% of the time. None of the follow-up survey responses indicating using test strips 100% of the time.

#### Overdose Risk Reduction Behaviors.

There was also a marked discrepancy between the daily reports and follow-up surveys in terms of the overdose risk reduction behaviors taken. The daily reports yielded a much higher proportion of participants (81.8%) indicating they had taken one or more risk reduction precautions after a positive fentanyl test compared to a much lower rate (45.5%) based on the follow-up survey responses.

#### Past-month Overdoses.

The number of participants experiencing an overdose in the past month, 27.3% (3 of 11 participants) was consistent between the experience sampling event-driven reporting form and the follow-up survey.

## Discussion and Conclusions

We conducted this pilot study to assess the feasibility of using one form of EMA, daily experience sampling, to collect information on fentanyl and benzodiazepine test strip use among opiate street drug users. Secondarily, we also wanted to compare the information obtained thereby with retrospectively collected data as is presently the norm in the DCS research literature. Our interpretation of the results is that daily experience sampling, while resource intensive, is feasible with this population, which included persons experiencing homelessness as well as persons with a co-occurring SMI, two issues that could preclude submitting daily DCS reports. Daily reports were submitted on 160 unique “person days” out of 252 possible days, yielding an overall response rate of 63.5%. This rate improves to 70% when two outliers who submitted fewer than 2 reports over the 21-day collection period are excluded.

Moreover, on reports where drug use for that day was indicated, just over half of the reports (53.3%) indicated testing had been done using the provided test strips, providing additional information on what overdose risk reduction steps had been taken and when. This result seems particularly important for advancing research on how best to implement DCS to reduce opioid-related overdoses. As noted in the introduction, experience sampling can provide more detail on the dynamic associations between test strip use, risk reduction behaviors, and overdoses compared with retrospective reports, which also have the drawback of various recall-related biases.

One of the potential associations revealed is that it makes sense from some users’ perspectives to not test a street drug prior to use on a given day if the drug being used has been tested previously; in this instance users likely calculate that further testing would provide no additional information. However, this may or may not be true as it is possible to have tested only a very small sample from one part of the purchased drug with fentanyl still present and in quantity in the remaining portion of the purchased drug. Additionally, not testing because the drug had been purchased from the same dealer – one of the main reasons participants gave for not using a test strip at all on a given sample – is also not a good strategy *ceteris paribus* given potential variations in the dealer’s drug supply and from batch to batch. Such detailed information can be used to counsel users on the importance of testing any newly purchased street drug regardless of the supplier or whether the drug has been previously tested.

Owing to the small sample size and that this was a feasibility study, we can’t draw strong inferences from the results in terms of the implications for providing and closely monitoring test strip use. For instance, we don’t know why reports were not submitted on 30% to 40% of all possible submission days. In retrospect, we should have included follow-up questions on this issue or programmed in a brief prompt the day after no report was received to find out the reason why. We suspect, however, given the high rate of reported test strip use on the daily surveys that were provided, days when a test strip was not used were under-reported. More information is needed to optimize the daily prompting schedule and question set to reduce participant burden while concurrently improving response rate and accuracy.

We also need to obtain more detailed information to understand the discrepancies between the daily reports and the retrospective data in terms of percent of time used fentanyl test strips and the overdose precautions taken. One possible explanation for at least part of the discrepancy between the retrospective and daily reports, is that the retrospective reports were based on the past-month whereas the daily reports were based on a 21-day period. In future studies, having both data collection methods covering the same time period could reduce the discrepancies we found.

Another explanation, as revealed by the daily reports, is the conditional nature of using test strips on the sample for a given day and taking overdose risk precautions conditional on the test results. It is likely that participants do not consider the conditional nature of the behavioral chain that leads from deciding to test a drug to deciding to take overdose risk precautions. Having retrospective questions that consider these behavioral contingencies might provide more accurate information.

### Limitations

Aside from the small sample size and the need to refine and validate the daily reporting forms and protocol, another limiting factor was that we recruited participants who had their own mobile phones for use. To include a broader sample that is likely more representative of the street drug using population, persons who do not have their own mobile phones would have to be included. Whether a person does or does not use their own phone is a potential factor to monitor in terms of the effect on response rates in a future study. Inclusion of persons with their own phones could reduce the daily reporting response rates beyond what we obtained owing to a higher probability of lost phones, etc.

We also paid participants to provide daily reports using a payment schedule that rewarded greater reporting frequency. This could have influenced or encouraged test strip use and/or reporting (by design) with the study results not accurately representing test strip use and risk reduction when a person is not on a payment schedule. As our focus was on persons reporting relatively frequent opioid use (3+ times weekly), we also do not know how the results would apply to less frequent users or to users of other street drugs such as cocaine or methamphetamine, who also need to test their drugs given that fentanyl is now being used broadly as an admixture with drugs other than opioids.

## Data Availability

The data set analyzed for the current study is available from the corresponding author on reasonable request.

## Key messages regarding feasibility

1. **What uncertainties existed regarding feasibility?** It has not been determined whether it is feasible to collect daily information on drug checking and opioid overdose risk reduction from street drug users using a mobile phone-based experience sampling application. Presently, most studies of drug checking using test strips rely on retrospective reports.
2. **What are the key feasibility findings?** Participants had a reporting rate of 60% to 70% in terms of the proportion of data collection days they submitted a drug checking report. They also indicated the experience sampling application was easy to use. We believe these findings support using experience sampling to collect patient reported outcome measures related to drug checking and overdose risk reduction.
3. **What are the implications of the feasibility study for designing the main study?** We believe they indicate we can use experience sampling in a larger study to better understand circumstances when a person does and does not test their street drugs and test different interventions to increase drug checking such as tailoring information to each user’s specific circumstances. Having established 21 days as a reasonable data collection period, we can also now move forward to assess collecting data over a longer period of time and experiment with data collection on randomly selected days/times to see if a similar response rate is obtained.

## List of abbreviations

DCS: drug checking services
DF: degrees of freedom
EMA: Ecological momentary assessment
MDMA: 3,4-methylenedioxy-methamphetamine (i.e., “Ecstasy”)
mEMA: mobile Ecological momentary assessment
OD: overdose/overdoses
OEND: opioid education and naloxone distribution
OROF: opioid-related overdoses and fatalities
SD: standard deviation
SSP: syringe service programs
